# Linagliptin Improves Insulin Sensitivity and Hepatic Steatosis in Diet-Induced Obesity

**DOI:** 10.1371/journal.pone.0038744

**Published:** 2012-06-22

**Authors:** Matthias Kern, Nora Klöting, Heiko G. Niessen, Leo Thomas, Detlef Stiller, Michael Mark, Thomas Klein, Matthias Blüher

**Affiliations:** 1 Department of Medicine, University of Leipzig, Leipzig, Germany; 2 IFB Obesity Diseases, Junior Research Group Animal Models, University of Leipzig, Leipzig, Germany; 3 Boehringer-Ingelheim Pharma GmbH & Co. KG, Biberach, Germany; University of Las Palmas de Gran Canaria, Spain

## Abstract

Linagliptin (tradjenta™) is a selective dipeptidyl peptidase-4 (DPP-4) inhibitor. DPP-4 inhibition attenuates insulin resistance and improves peripheral glucose utilization in humans. However, the effects of chronic DPP-4 inhibition on insulin sensitivity are not known. The effects of long-term treatment (3–4 weeks) with 3 mg/kg/day or 30 mg/kg/day linagliptin on insulin sensitivity and liver fat content were determined in diet-induced obese C57BL/6 mice. Chow-fed animals served as controls. DPP-4 activity was significantly inhibited (67–89%) by linagliptin (*P*<0.001). Following an oral glucose tolerance test, blood glucose concentrations (measured as area under the curve) were significantly suppressed after treatment with 3 mg/kg/day (–16.5% to –20.3%; *P*<0.01) or 30 mg/kg/day (–14.5% to –26.4%; *P*<0.05) linagliptin (both *P*<0.01). Liver fat content was significantly reduced by linagliptin in a dose-dependent manner (both doses *P*<0.001). Diet-induced obese mice treated for 4 weeks with 3 mg/kg/day or 30 mg/kg/day linagliptin had significantly improved glycated hemoglobin compared with vehicle (both *P*<0.001). Significant dose-dependent improvements in glucose disposal rates were observed during the steady state of the euglycemic–hyperinsulinemic clamp: 27.3 mg/kg/minute and 32.2 mg/kg/minute in the 3 mg/kg/day and 30 mg/kg/day linagliptin groups, respectively; compared with 20.9 mg/kg/minute with vehicle (*P*<0.001). Hepatic glucose production was significantly suppressed during the clamp: 4.7 mg/kg/minute and 2.1 mg/kg/minute in the 3 mg/kg/day and 30 mg/kg/day linagliptin groups, respectively; compared with 12.5 mg/kg/minute with vehicle (*P*<0.001). In addition, 30 mg/kg/day linagliptin treatment resulted in a significantly reduced number of macrophages infiltrating adipose tissue (*P*<0.05). Linagliptin treatment also decreased liver expression of *PTP1B*, *SOCS3*, *SREBP1c*, *SCD-1* and *FAS* (*P*<0.05). Other tissues like muscle, heart and kidney were not significantly affected by the insulin sensitizing effect of linagliptin. Long-term linagliptin treatment reduced liver fat content in animals with diet-induced hepatic steatosis and insulin resistance, and may account for improved insulin sensitivity.

## Introduction

Nutrient intake stimulates the release of the incretin hormones glucose-dependent insulinotropic polypeptide (GIP) and glucagon-like peptide-1 (GLP-1) into the circulation [1]–[3]. The important functions of both these incretins include potentiating glucose-dependent insulin secretion from pancreatic β-cells and inhibiting glucagon secretion, which in turn reduces hepatic gluconeogenesis [2], [4], [5]. The ‘incretin effect’ is significantly reduced in patients with type 2 diabetes mellitus (T2DM) and contributes to impaired insulin secretion and chronic hyperglycemia [6], [7]. Under normal physiologic conditions, dipeptidyl peptidase-4 (DPP-4) rapidly degrades GIP and GLP-1 [2], [4]. Thus, the inhibition of DPP-4 is a therapeutic option to extend the half-life of endogenous GIP and GLP-1, and lower hyperglycemia in patients with T2DM. Several DPP-4 inhibitors have demonstrated a favorable safety and tolerability profile in clinical studies of patients with T2DM [2], [8]. Other beneficial effects observed in animal and *in vitro* studies include inhibition of β-cell apoptosis [9] caused by glucotoxicity and/or lipotoxicity [10], modulation of β-cell mass and islet cell proliferation [11], [12], and differentiation of pancreatic ductal cells into insulin-producing cells [13].

Linagliptin, a xanthine-based, highly potent and long-acting non-peptidomimetic DPP-4 inhibitor, was recently approved in the United States for the treatment of T2DM [14], [15]. In both animal and *in vitro* studies, linagliptin demonstrated a greater inhibition of DPP-4 than alogliptin, saxagliptin, sitagliptin or vildagliptin [15]. After absorption, linagliptin binds to plasma proteins in a concentration-dependent manner, giving the drug a nonlinear pharmacokinetic profile [16]. Unlike other DPP-4 inhibitors that are cleared by the kidneys, linagliptin is mainly excreted in the feces [17], [18]. The high therapeutic index and placebo-like safety profile of linagliptin support once-daily dosing in patients with T2DM, with no requirement for dose adjustment in patients with declining renal function [4], [19].

A pilot study in 16 patients with T2DM reported improved insulin sensitivity after 6 weeks of treatment with the DPP-4 inhibitor vildagliptin [20]. In another study, sitagliptin prevented the development of metabolic and hormonal disturbances, and increased β-cell apoptosis and liver steatosis induced by a fructose-rich diet in normal rats [21]. Long-term treatment with the DPP-4 inhibitor P32/98 caused sustained improvements in glucose tolerance, insulin sensitivity, hyperinsulinemia and β-cell glucose responsiveness in Vancouver diabetic fatty (fa/fa) Zucker rats [22] and in glucokinase haploinsufficient diabetic mice [23]. We therefore tested the hypothesis that long-term (3–4 weeks) linagliptin treatment in mice with diet-induced obesity (DIO) would improve insulin sensitivity. In addition, we investigated the effects of linagliptin treatment on glycemic control and liver fat content.

**Table 1:** Study design.

Study	Duration of HFD (months)	Duration of linagliptintreatment (weeks)	Animals per group (*n*)	Outcome measures
1	2	4	9	Liver fat content (MRS) OGTT
2	3	4	15	Insulin sensitivity (clamp) OGTT
3	3	3	9	Liver fat content (MRS) OGTT
4	4	4	9	Liver fat content (MRS) OGTT

Female C57BL/6N mice were fed with a HFD (60% calories from fat) or a chow diet (10% calories from fat) for either 2, 3 or 4 months. Therapeutic intervention was initiated following the feeding period either for 3 or 4 additional weeks. Linagliptin was administered orally daily in doses of 3 mg/kg or 30 mg/kg in 0.5% Natrosol (or Natrosol alone as vehicle control). One group of chow-fed mice (*n* = 15) was used as comparators for all studies.

HFD, high-fat diet; MRS, magnetic resonance spectroscopy; OGTT, oral glucose tolerance test.

## Methods

### Experimental Animals and Study Design

Ten-week-old female C57BL/6N mice (*n* = 57) were purchased from Charles River (Boston, MA). The study design comprised four individual studies (Table 1) in which obesity was induced by feeding the mice a high-fat diet (60 kcal% fat; Research Diets D12492, Open Source Diets, New Brunswick, NJ) for 2, 3 or 4 months, depending on the study protocol. Chow-fed mice (*n* = 15) were used as controls for all the studies. Therapeutic intervention with linagliptin or vehicle was initiated after the high-fat feeding period for 3 or 4 additional weeks. Linagliptin (3 or 30 mg/kg in 0.5% Natrosol) was administered orally once-daily between 0800 and 0900 using a cannula; Natrosol alone was used as vehicle control. Clamp studies and magnetic resonance spectroscopy (MRS) studies used 15 and nine animals per group, respectively. Fed plasma glucose concentrations were measured once weekly before treatment. Experiments were performed in accordance with the rules for animal care of the local government authorities and were approved by the animal care and use committee of Leipzig University as well as by the animal care committee of the Bezirksregierung Leipzig, Germany (Approval ID: TVV 27/08).

**Table 2:** Influence of linagliptin treatment on DPP-4 activity, serum GLP-1 concentrations, serum glucose during OGTT and liver TG content.

	DPP-4 inhibition(% of vehicle treated)	GLP-1 (pM)	Glucose AUC_0–120 min_ (% reduction compared with vehicle treated)	Liver TG (µg/mg)
*Study 1 (2 months HFD)*				
Vehicle	NA	8.8±2.7	NA	31.3±2.5
Linagliptin 3 mg/kg/day	67***	39.9±23.6***	–16.5**	31.4±1.3
Linagliptin 30 mg/kg/day	79***	46.2±5.9***	–14.5*	25.13±1.4*
Chow fed	0.7 (NS)	12.9±3.3 (NS)	–25.0***	15.8±1.0***
*Study 3 (3 months HFD)*				
Vehicle	NA	ND	NA	NP
Linagliptin 3 mg/kg/day	80***	45.0±10.0	–20.3**	NP
Linagliptin 30 mg/kg/day	89***	56.2±8.4	–26.4*	NP
Chow fed	3	9.1±1.6	–25.5***	NP
*Study 4 (4 months HFD)*				
Vehicle	NA	2.3±0.4	NP	58.8±9.1
Linagliptin 3 mg/kg/day	78***	26.8±2.5***	NP	41.2±2.7
Linagliptin 30 mg/kg/day	84***	38.7±2.5***	NP	42.5±2.8
Chow fed	16	4.8±1.3	NP	21.8±2.8***

Values are given as mean ± SEM. *P<0.05; **P<0.01; ***P<0.001 compared with vehicle-treated mice.

AUC, area under the curve; DPP-4, dipeptidyl peptidase-4; GLP-1, glucagon-like peptide-1; HFD, high-fat diet; NA, not applicable; ND, not detectable; NP, not performed; NS, not significant; OGTT, oral glucose tolerance test; TG, triglycerides.

### Measurement of DPP-4 Activity

Aliquots of 20 µl plasma in EDTA were diluted with 30 µl of DPP-4 assay buffer (100 mM Tris, 100 mM NaCl, adjusted to pH 7.8 with HCl) and mixed with 50 µl of substrate, *H*-Ala-Pro-7-amido-4-trifluoromethylcoumarin (Bachem, Bubendorf, Switzerland) to a final concentration of 100 µM. The reaction was incubated at room temperature for 10 minutes and DPP-4 activity measured as fluorescence emission at 535 nm with excitation at 405 nm using a Wallac, Victor™ 1420 Multilabel Counter (PerkinElmer, Waltham, MA).

**Table 3:** Effects of 4 weeks linagliptin treatment in C57BL/6N mice after 3 months of HFD (study 2; *n* = 15 per experimental group) on GLP-1 serum concentrations, food intake, body weight, fed glucose levels, HbA1c and liver triglyceride content.

	GLP-1 (pM)	Food intake(g/day)	Body weight (g)	Glucose (mM)	HbA1c (%)	Liver TG (µg/mg)
Vehicle	ND	2.8±0.3	44.4±0.5	6.2±0.1	4.6±0.03	60.7±3.2
Linagliptin 3 mg/kg/day	18.4±4.0	3.1±0.2	44.3±0.7	5.8±0.8***	4.3±0.03***	47.9±2.4***
Linagliptin 30 mg/kg/day	26.4±3.7	3.1±0.4	44.7±1.6	5.7±0.07***	4.3±0.03***	39.1±2.4***
Chow fed	7.5±3.4	4.0±0.3	25.2±0.5	5.7±0.08***	4.3±0.03***	14.3±0.9***

Values are given as mean ± SEM. ***P<0.001 compared with vehicle-treated DIO mice.

GLP-1, glucagon-like peptide-1; HbA1c, glycated hemoglobin; HFD, high-fat diet; ND, not detectable; TG, triglycerides.

### Oral glucose Tolerance Tests

Oral glucose tolerance tests (OGTT) were performed after an overnight fast for 16 hours. Animals were orally loaded with 2 g/kg body weight glucose and tail vein blood was collected at 0 (baseline), 15, 30, 60 and 120 minutes following challenge. Blood glucose concentration was measured with a glucometer (OneTouch® Ultra®; Lifescan, Milpitas, CA).

**Figure 1:**
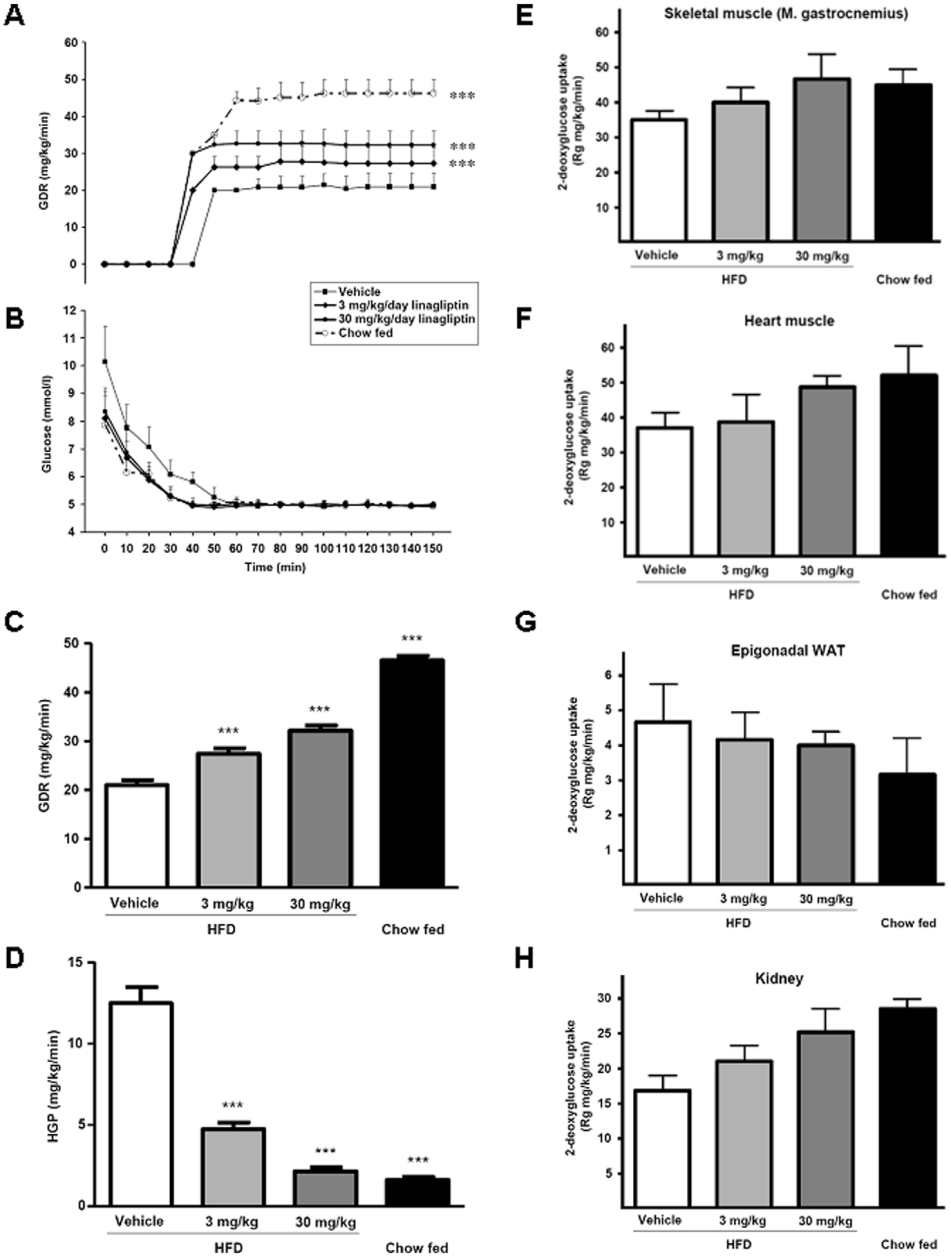
Effect of linagliptin on insulin sensitivity determined by euglycemic–hyperinsulinemic clamps in diet-induced obesity (DIO) mice. C57BL/6 mice were fed a high-fat diet (HFD) or chow diet for 3 months followed by treatment with linagliptin (3 mg/kg/day or 30 mg/kg/day) or placebo. Four weeks after treatment initiation, euglycemic–hyperinsulinemic clamps were performed to measure glucose disposal rate (GDR) (A), dynamic of blood glucose concentrations (B), and GDR and hepatic glucose production (HGP) during the steady-state period of the euglycemic–hyperinsulinemic clamp (C and D). (E–H) Tissue specific uptake of 2-deoxy-*d*-[1-^14^C]glucose into gastrocnemius skeletal muscle (E), heart muscle (F), perigonadal white adipose tissue (WAT) (G) and kidney (H). Blood glucose levels were determined every 10 minutes (B-Glucose Analyzer; HemoCue AB, Ängelholm, Sweden). Values are given as mean ± SD. ***P<0.001 compared with DIO vehicle-treated group.

### Euglycemic–hyperinsulinemic Clamp Studies (Study 2)

For catheter implantation, mice were anesthetized in the fed state 4 weeks after the start of treatment with an intraperitoneal injection of 240 mg/kg body weight Avertin^®^ (2,2,2 Tribromoethanol, 2-methyl-2-butanol; Sigma Aldrich, Hamburg, Germany).

After loss of pedal reflex was confirmed, a catheter (MicroRenathane® tubing, MRE 025; Braintree Scientific Inc., Braintree, MA) was inserted into the right internal jugular vein and advanced to the superior vena cava [24]. The vein was then ligated distally. The catheter was filled with 100 µl of NaCl/heparin sulfate solution to prevent clotting. The end of the catheter was tunneled to the supra scapular region. Mice were administered intraperitoneal injections of 1 ml saline containing 15 mg/g body weight of tramadol and placed on a heating pad to facilitate recovery.

**Figure 2:**
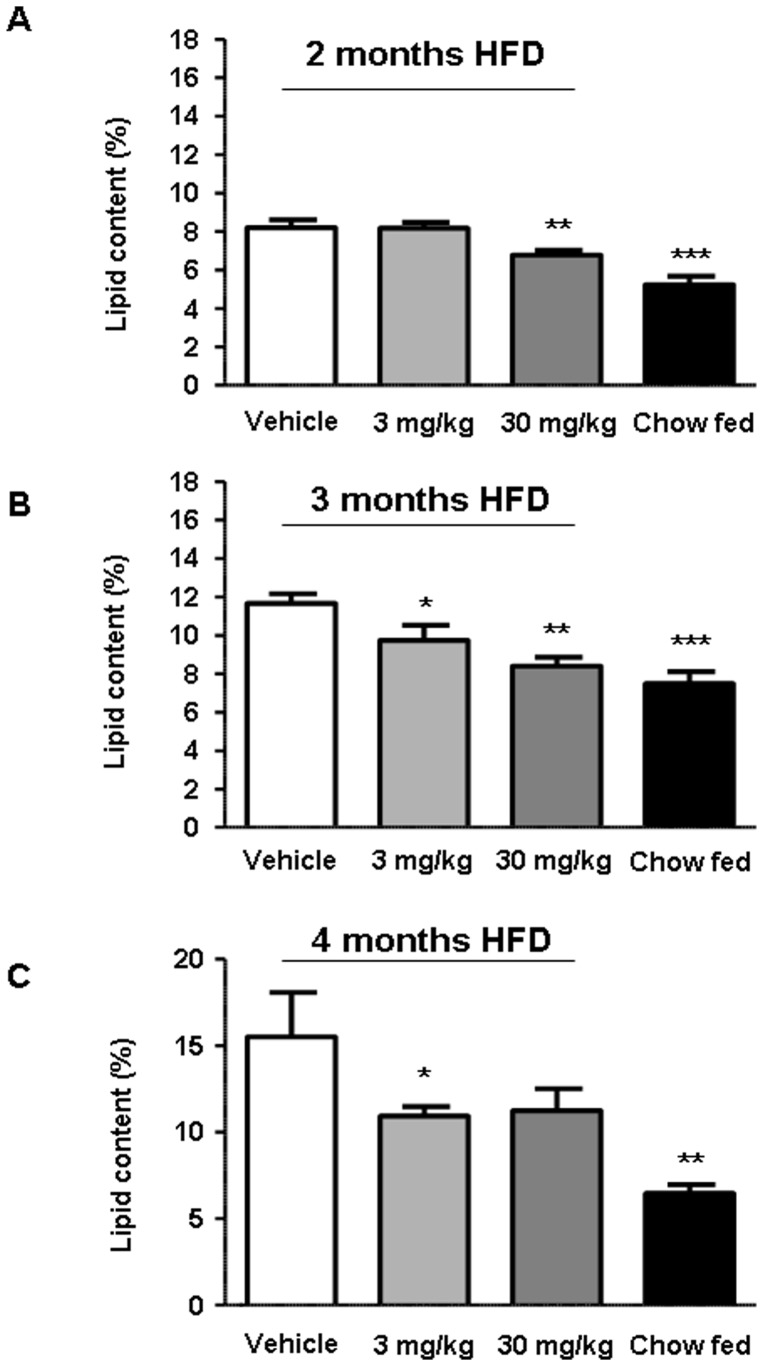
Effect of linagliptin treatment on liver lipid content in diet-induced obesity (DIO) mice. C57BL/6 mice were fed a high-fat diet (HFD) for either 2 months and treated with linagliptin (3 mg/kg/day or 30 mg/kg/day) or placebo for an additional 4 weeks (A), HFD for 3 months and treated with linagliptin (3 mg/kg/day or 30 mg/kg/day) or placebo for an additional 3 weeks (B), or HFD for over 4 months and treated with linagliptin (3 mg/kg/day or 30 mg/kg/day) or placebo for an additional 4 weeks (C). Liver lipid content was determined using magnetic resonance spectroscopy. Duration of HFD is displayed for the individual graphs. Values are given as mean ± SEM. *P<0.05; **P<0.01; ***P<0.001.

**Figure 3:**
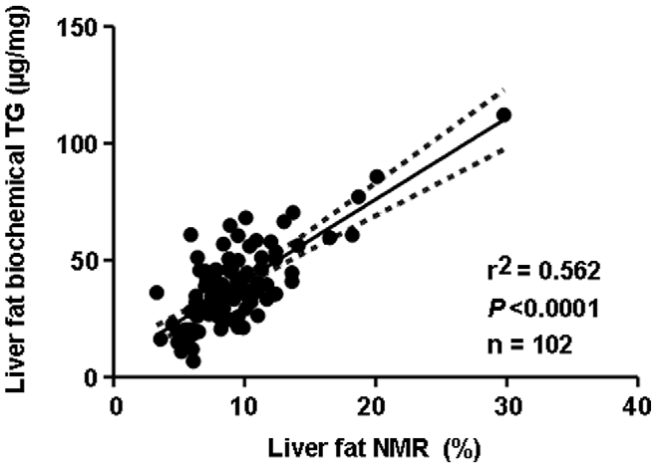
Correlation of liver triglyceride content detected with enzymatic assays and relative liver lipid content measured with magnetic resonance spectroscopy.

Euglycemic–hyperinsulinemic clamps were performed on animals in the fed state 3 days after catheter implantation. After a 5-mCi bolus injection of d-[3-^3^H]glucose (Amersham Biosciences, Little Chalfont, UK), the tracer was infused continuously (0.05 mCi/minute) for the duration of the experiment. Baseline parameters were determined using a 50-µl aliquot of blood collected at the end of the 40-minute run-in period. To minimize blood loss, red blood cells were collected by centrifugation, resuspended in saline and re-infused. A bolus of insulin (40 mU/g; Actrapid 40U, Novo Nordisk, Copenhagen, Denmark) solution containing 0.1% bovine serum albumin (BSA; Sigma-Aldrich, Hamburg, Germany) was injected followed by infusion at a fixed rate (4 mU/g/minute). Blood glucose levels were determined every 10 minutes (B-Glucose Analyzer; HemoCue AB, Ängelholm, Sweden). Physiological blood glucose concentrations (between 120 and 150 mg/dl) were maintained by adjusting infusion of a 20% glucose (DeltaSelect, Rimbach, Germany) solution. Approximately 60 minutes before steady state was achieved, a bolus of 2-deoxy-d-[1-^14^C]glucose (10 mCi; Amersham Biosciences, Little Chalfont, UK) was infused. Steady state was ascertained when glucose measurements were constant for ≥30 minutes at a fixed glucose infusion rate and was achieved within 120–150 minutes. During the clamp experiment, 5-µl blood samples were collected after infusion of the 2-deoxy-d-[1-^14^C]glucose at 0 and 5 minutes, and then at 10-minute intervals thereafter, until steady state was reached. Once steady state had been reached, 50-µl blood samples were collected for the measurement of steady-state parameters. At the end of the experiment, mice were euthanized with an Avertin® overdose, and epigonadal adipose tissue, subcutaneous adipose tissue, skeletal muscle, liver, brain, kidney and heart were taken and stored for biochemical/molecular analyses. Plasma [3-^3^H]glucose and deoxy-[1-^14^C]glucose radioactivity of baseline and steady-state samples were determined as described [24]. Glucose disposal rate (GDR), in mg/kg/minute, was calculated as the rate of tracer infusion (dpm/minute) divided by the plasma glucose-specific activity (dpm/mg) corrected for body weight. Hepatic glucose production (HGP), in mg/kg/min, was calculated as the difference between the rates of glucose appearance and glucose infusion.

**Figure 4:**
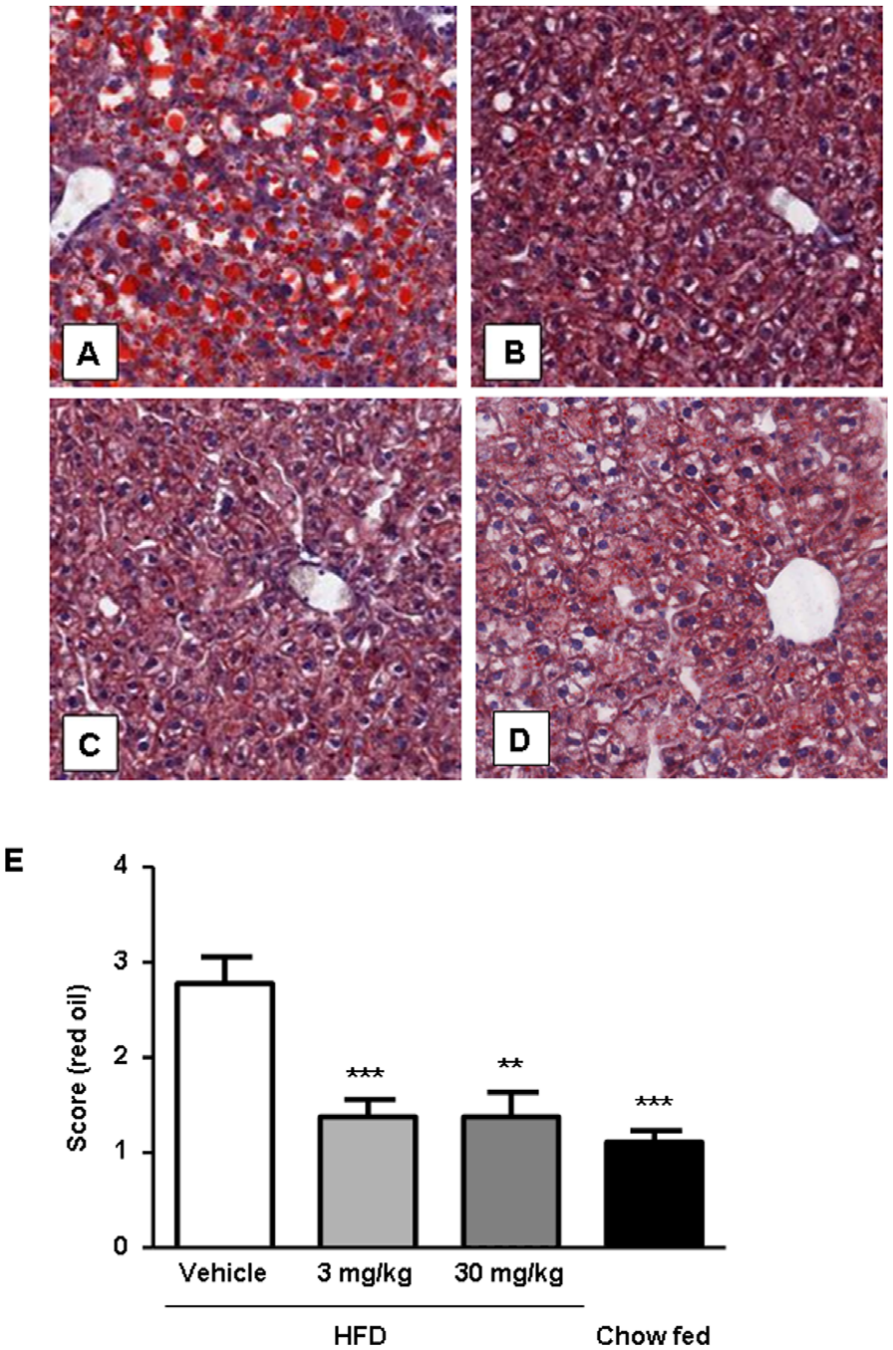
Evaluation of effect of linagliptin on hepatic steatosis. Oil Red O staining of liver specimens from diet-induced obesity (DIO) mice treated with vehicle (A), DIO mice treated with linagliptin 3 mg/kg/day (B), DIO mice treated with linagliptin 30 mg/kg/day (C) and chow-fed mice (D). Quantification of Oil Red O staining was performed by histologic scoring (*n* = 9 animals per group) (E). Data are shown as mean ± SEM. **P<0.01; ***P<0.001.

### Magnetic Resonance Spectroscopy (MRS) of Liver

Mice were anesthetized by continuous inhalation of 2% isoflurane (Forene, Abbott, Ludwigshafen, Germany) in a N_2_O:O_2_ (70∶30 v:v) gas mixture. Liver lipid content was measured by MRS using a Bruker BioSpec® 47/40 scanner (Bruker BioSpin, Ettlingen, Germany) equipped with a BGA12 gradient coil system. A volume coil was used for excitation and a surface coil for signal reception. For anatomical orientation, a pilot scan containing horizontal and axial images was acquired using a gradient-echo pulse sequence with the following parameters: TR 135 ms; TE 3.5 ms; field of view 45×45 mm^2^; matrix 128^2^ and slice thickness 1.75 mm. Based on the pilot scan, a voxel of interest (3×3×3 mm^3^) was placed in the left ventral part of the liver. Liver lipids in the voxel of interest were measured using a point-resolved spectroscopy (PRESS) sequence with the following parameters: TR 1050 ms; TE 20 ms; number of averages 32 and digital resolution 1024 data points. Since the magnetic resonance spectrum of a liver voxel contains signals from water and lipids, liver lipid content was standardized and expressed as the ratio (in %) between lipid signals and water signal. Data were analyzed with commercially available LCModel software [25].

**Figure 5:**
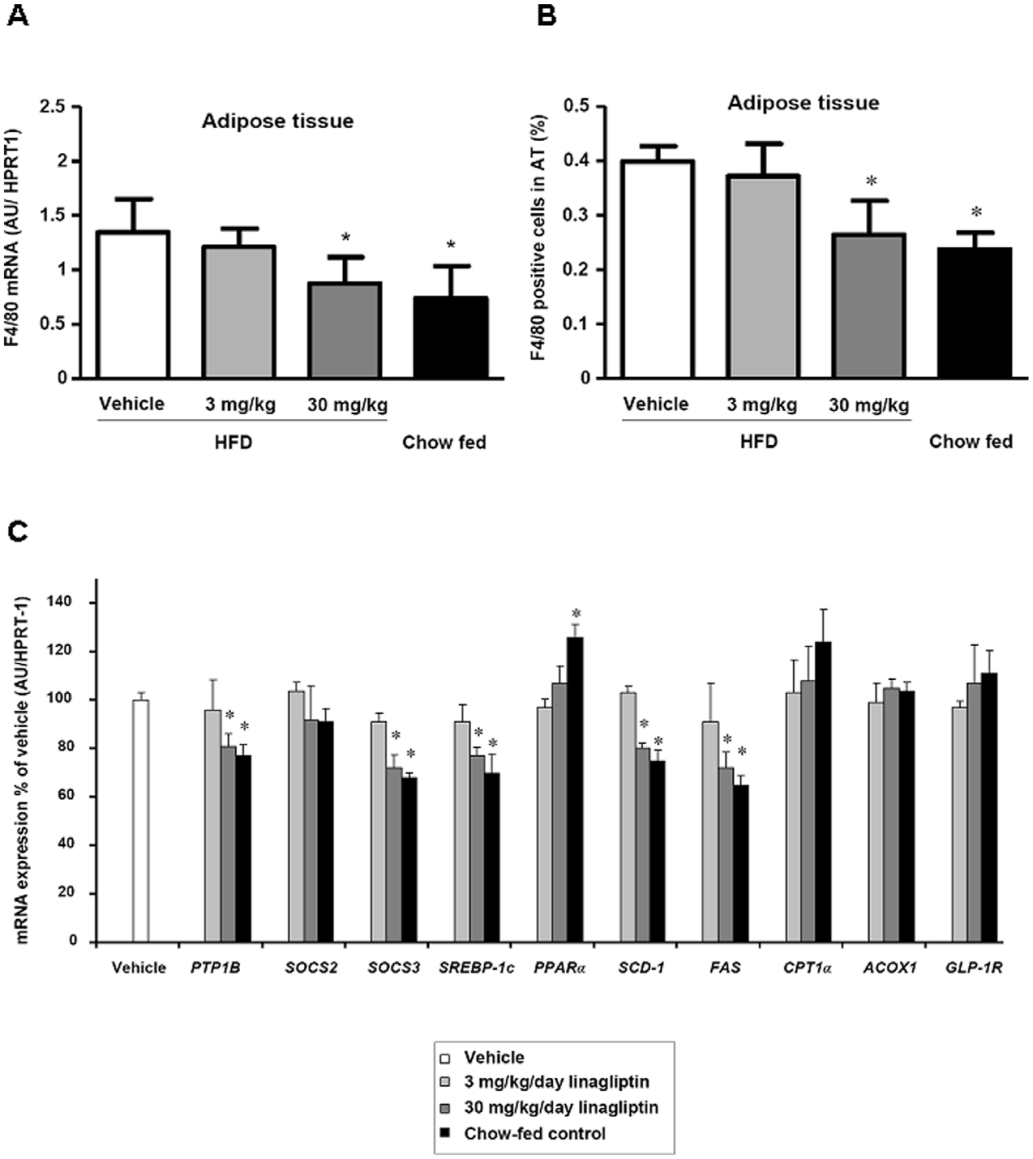
Effects of linagliptin on macrophage infiltration into adipose tissue and on liver mRNA expression of key molecules in liver metabolism and inflammation. (A) mRNA expression of F4/80 and (B) number of F4/80 positive cells in epigonadal adipose tissue of C57BL/6 mice which were fed a high-fat diet (HFD) or chow diet for 3 months followed by treatment with linagliptin (3 mg/kg/day or 30 mg/kg/day) or placebo for 4 weeks. (C) mRNA expression of *protein tyrosine phosphatase 1B (PTP1B), suppressor of cytokine signaling 2 (SOCS2), suppressor of cytokine signaling 3 (SOCS3), sterol regulatory element–binding protein-1c* (*SREBP1c*), *peroxisome proliferator–activated receptor-α* (*PPARα*), *stearoyl-CoA desaturase-1* (*SCD-1*), *fatty acid synthase (FAS), carnitine palmitoyl transferase 1α* (*CPT1α*), *acyl-CoA oxidase 1* (*ACOX1*), and *GLP-1 receptor* in the liver of C57BL/6 mice which were fed a high-fat diet (HFD) or chow diet for 3 months followed by treatment with linagliptin (3 mg/kg/day or 30 mg/kg/day) or placebo for 4 weeks. mRNA expression was calculated relative to the mRNA expression of *hypoxanthine phosphoribosyltransferase 1 (HPRT-1).* Data are shown as mean ± SEM. *P<0.05 vs. vehicle.

### Liver Histology

Histologic analyses of liver were performed after liver tissue samples were fixed at room temperature in 4% formaldehyde and embedded in paraffin. Five-micrometer sections were mounted on glass slides, deparaffinized in xylol, and stained for hematoxylin and eosin. Additional Oil Red O staining of the histologic slides was used for the localization of lipids that exist as neutral lipids and fatty acids. Quantification of Oil Red O staining was performed by histologic scoring (*n* = 9 animals per group).

**Figure 6:**
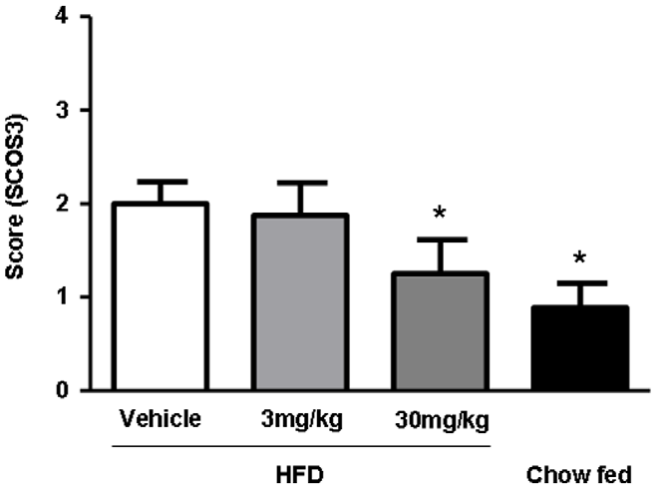
Effects of linagliptin on liver Suppressor of cytokine signaling 3 (SOCS3) protein expression. SOCS3 protein staining of liver specimens from diet-induced obesity (DIO) mice treated with vehicle, linagliptin 3 mg/kg/day (B), linagliptin 30 mg/kg/day (C) and chow-fed mice (D). Quantification of staining was performed by histologic scoring (*n* = 9 animals per group). Data are shown as mean ± SEM. *P<0.05.

SOCS3 immunohistochemisty was performed with Abcam (ab16030) antibody in 1/200 dilution of pepsin pretreated slides. Scoring was done by an experienced pathologist in a blinded manner using a scoring scale from 0–4 (no, minimal, mild, moderate, severe).

### Measurement of mRNA Expression in Adipose Tissue and Liver (Study 2)

Total RNA was isolated from epigonadal adipose tissue and liver using TRIzol (Life Technologies, Grand Island, NY) and 1 µg RNA was reverse transcribed with standard reagents (Life Technologies, Grand Island, NY). cDNA was prepared using the Taqman reverse transcriptase kit (Applied Biosystems, Darmstadt, Germany). From each reverse transcription polymerase chain reaction (PCR) 2 µl were amplified in a 26 µl PCR reaction using Brilliant SYBR Green QPCR Core Reagent Kit (Stratagene, La Jolla, CA). The following primer pairs were used: *sterol regulatory element–binding protein-1c* (*SREBP1c*), 5′ ACAGAGCTTCCGGCCTGCTA 3′ (sense) and 5′ CCGAGCTG TGGCCTCATGTA 3′ (antisense); *peroxisome proliferator–activated receptor-α* (*PPARα*), 5′ TCAAGG TGTGGCCCAAG GTTA 3′ (sense) and 5′ CGAATGTTCTCAGAAGCCAGCT 3′ (antisense); *stearoyl-CoA desaturase-1* (*SCD-1*), 5′ GCCTGT ACGGGATCATACTGGTTC 3′ (sense) and 5′ CAGAGC GCTGGT CATGTAGTAGA3′ (antisense); *carnitine palmitoyl transferase 1α* (*CPT1α*), 5′ AAGC CTTTGGGTG GTGGATATGTGA3′ (sense) and 5′ TGGAACTGGTGGCCAAT GAG3′ (antisense)*; acyl-CoA oxidase 1* (*ACOX1*), 5′ CAGCGTTACGAGGTGGCTGTTA3′ (sense) and 5′ TGCCCAA GTGAAGGTCC AAAG 3′ (antisense) and *GLP-1 receptor*, 5′ ATGGTGG CTATCCTGTAD TGCTTTTG 3′ (sense) and 5′ GCTGCTGGTGGGACACTTGA 3′ (antisense). Expression of mRNAs were quantified by using the second derivative maximum method of the TaqMan software (Applied Biosystems, Darmstadt, Germany), determining the crossing points of individual samples by an algorithm which identifies the first turning point of the fluorescence curve. Amplification of specific transcripts was confirmed by melting curve profiles (cooling the sample to 68°C and heating slowly to 95°C with measurement of fluorescence) at the end of each PCR. Expression of *hypoxanthine phosphoribosyltransferase 1, F4/80, protein tyrosine phosphatase 1B (PTP1B), fatty acid synthase (FAS), suppressor of cytokine signaling 2 (SOCS2)* and *suppressor of cytokine signaling 3 (SOCS3)* was measured using TaqMan Gene Expression Assays (Applied Biosystems, Darmstadt, Germany).

### Measurement of Other Parameters

Blood glucose and glycated hemoglobin (HbA1c) values were determined from 5-µl whole venous blood samples using an automated glucose monitor (HITADO Blutglukose Analyzer Super GL, Münster, Germany). Fasting glucose levels and capillary blood glucose measurements were determined from 1 µl of tail vein samples using a Freestyle Mini Analyzer (Abbott, Berlin, Germany). Active GLP-1 was detected as previously reported [15].

### Statistical Analyses

Data are means ± standard deviation unless stated otherwise. Before statistical analysis, non-normally distributed parameters were logarithmically transformed to approximate a normal distribution. The following statistical tests were performed using SPSS version 12.0 (Chicago, IL): unpaired Student’s *t*-test; ANOVA and Pearson’s simple correlation. Linear relationships were assessed by least square regression analysis. *P* values of <0.05 were considered statistically significant.

## Results

### Effects of Long-term Linagliptin Treatment on Serum DPP-4 Activity and GLP-1 Levels

DPP-4 activity was significantly reduced and GLP-1 levels were significantly increased in all the studies at both linagliptin doses, compared with treatment with vehicle alone (*P*<0.001 for all comparisons; Table 2).

### Effects of Linagliptin on Glucose Metabolism

Treatment of DIO mice for 4 weeks with either dose of linagliptin (study 2) significantly lowered fed blood glucose concentrations and HbA1c (both *P*<0.001) compared with treatment with vehicle (Table 3). In studies 1 and 3, blood glucose levels during oral glucose tolerance tests measured as area under the curve were significantly suppressed compared with vehicle: –16.5% and –20.3% with linagliptin 3 mg/kg/day (*P*<0.01, both studies); and –14.5% and –26.4% with linagliptin 30 mg/kg/day (*P*<0.05, both studies; Table 2).

### Effect of Linagliptin on Insulin Sensitivity

Significant dose-dependent increases were seen in GDR during the steady-state period of the euglycemic–hyperinsulinemic clamp in the linagliptin-treated groups (27.3 mg/kg/minute and 32.2 mg/kg/minute in the 3 mg/kg/day and 30 mg/kg/day groups, respectively), compared with vehicle-treated DIO mice (20.9 mg/kg/minute; *P*<0.001 for both comparisons; Figure 1A–C). Chow-fed mice displayed still higher GDRs (46.2 mg/kg/minute) compared with vehicle-treated DIO mice (*P*<0.001; Figure 1C). Insulin-mediated suppression of HGP was significantly higher in both linagliptin-treated groups compared with vehicle-treated DIO mice during the steady-state period of the clamp (both *P*<0.001; Figure 1D). A notable finding was that the suppression of HGP in DIO mice treated with linagliptin 30 mg/kg/day was not statistically different from that of chow-fed mice (Figure 1D). Linagliptin treatment did not affect food intake or body weight in DIO mice (Table 3). There was a dose-dependent, but non-significant trend for higher 2-deoxy-d-[1-^14^C]glucose concentrations in the skeletal muscle, heart and kidneys of DIO mice treated with linagliptin compared with vehicle, whereas no such trend was observed in adipose tissue (Figure 1E–H).

### Effect of Linagliptin on Liver Lipid Content

Liver fat was detected by MRS (studies 1, 3 and 4; Figure 2) and with biochemical methods (studies 1, 3 and 4; Table 2 and study 2; Table 3). Additionally, liver fat content was evaluated by histologic examination of Oil Red O-stained specimens from study 4. Treatment of DIO mice with linagliptin reduced liver lipid content at 2, 3 and 4 months, with linagliptin 30 mg/kg/day significantly reducing liver lipid content (*P*<0.01) after 2 and 3 months of high-fat diet feeding (Figure 2). As expected, the chow-fed group always had the lowest liver lipid content independent of the study protocol (Figure 2). There was a highly significant correlation (*r* ^2^ = 0.56; *P*<0.001) between data obtained by MRS and biochemical triglyceride detection (Figure 3). Linagliptin-mediated reductions in liver triglyceride content were also significantly correlated with improvement in blood glucose (*r* ^2^ = 0.2, *P*<0.01) and HbA1c (*r* ^2^ = 0.18; *P*<0.01). Oil Red O-stained specimens from study 4 provided visual evidence of linagliptin-mediated liver fat reduction (Figure 4A–D), while quantification by histologic scoring revealed significant decreases in liver fat at both doses of linagliptin (3 mg/kg/day, *P*<0.001; and 30 mg/kg/day, *P*<0.01) compared with vehicle treatment (Fig. 4E).

### Effect of Linagliptin on Macrophage Infiltration into Adipose Tissue

Linagliptin treatment had no effect on mean adipocyte size in the epigonadal fat depot. In mice treated with 30 mg/kg/day linagliptin (as well as in chow-fed controls), a significantly lower (*P*<0.05) expression of the macrophage marker F4/80 was found compared with vehicle treatment (Figure 5A). These differences reflect a lower number of macrophages in the 30 mg/kg/day linagliptin treatment and chow-fed groups (Figure 5B).

### Effect of Linagliptin on mRNA Expression of Key Molecules in Liver Metabolism and Inflammation

Linagliptin treatment had a significant effect on the mRNA expression of several genes involved in the inflammatory response, fatty acid synthesis and oxidation. In parallel with reduced liver fat content, significantly decreased expression (*P*<0.05) of *PTP1B, SOCS3, SREBP1c, SCD1* and *FAS* with 30 mg/kg/day linagliptin treatment (Figure 5C) was found. There was no significant effect of the 3 mg/kg/day linagliptin dose on the expression of these genes. By contrast, linagliptin treatment had no effect on SOCS2, PPARα, CPT1α, ACOX1 or GLP-1 receptor mRNA expression (Figure 5C).

In addition, we performed immunohistochemistry for SOCS3 protein in the liver. The immunohistochemical staining for SOCS3 revealed a minimal to moderate, cytoplasmic staining of liver cells in a diffuse pattern. It was most severe in the vehicle group and showed a progessive decrease in linagliptin groups of 3 mg/kg and 30 mg/kg as well as chow treated animals (Figure 6). Following quantification the latter two groups were also significantly reduced. These data were in accordance to the results obtained for mRNA levels of SOCS3 (Figure 5C).

## Discussion

Linagliptin, a DPP-4 inhibitor, shows highly selective, potent, dose-dependent inhibition of the enzyme with >80% inhibition maintained throughout the 24-hour dosing interval [26]. In addition, linagliptin has a favorable pharmacokinetic profile as it is eliminated predominantly in the feces [4], [19]. Linagliptin is more effective than placebo in improving glycemic control both as monotherapy [27], [28] and in combination therapy with other oral agents, e.g. with pioglitazone [29]. Recently, it has been suggested that linagliptin may improve insulin sensitivity [29]. Furthermore, in a recent clinical pilot study, the DPP-4 inhibitor vildagliptin improved peripheral glucose utilization [20]. In the present study, we investigated the effects of 3 or 4 weeks of linagliptin treatment on DIO mice with respect to glucose metabolism and hepatic steatosis.

The key finding of our study is that linagliptin caused a dose-dependent improvement in insulin sensitivity in DIO mice as evaluated by GDR and insulin-mediated suppression of HGP. In addition, linagliptin reversed liver triglyceride content and improved hepatic steatosis in a dose-dependent manner, which correlated with improvements in blood glucose concentration and HbA1c. These data were consistently obtained, irrespective of the duration of high-fat diet preconditioning or linagliptin treatment. In all studies, linagliptin treatment significantly reduced DPP-4 activity and increased serum GLP-1 levels.

The improvement of insulin sensitivity in response to linagliptin treatment was a somewhat unanticipated finding. The main mechanisms by which DPP-4 inhibition lowers glucose levels are by improving glucose-dependent insulin secretion and reducing postprandial glucagon secretion [1]–[3]. A direct effect of either GLP-1 or GIP on glucose uptake cannot be excluded. In either case, it is possible that improved glycemia may reduce glucose toxicity-associated insulin resistance and thereby increase insulin sensitivity. However, while linagliptin treatment significantly reduced HbA1c in DIO mice, it was already low in vehicle-treated mice at 4.6%. Previously, the effects of glucose toxicity on insulin resistance have been previously reported with much larger reductions in glucose levels [30].

Another possible mechanism underlying linagliptin-induced improvements in glucose disposal is reduced lipotoxicity. Liver fat content correlates with the metabolic syndrome independent of obesity and may increase the risk for T2DM and atherosclerosis [30], [31]. Fat can accumulate in the liver through excess dietary fat (DIO mouse model), increased delivery of free fatty acids to the liver, inadequate fatty acid oxidation and increased *de novo* lipogenesis [31]. Stored triglycerides in muscle and liver may be reduced through reduction in fasting free fatty acid flux. However, the effects of linagliptin on free fatty acid flux or lipolysis were not tested and will require further investigation. Moderately hypocaloric, reduced-fat diets can decrease hepatic lipid content by approximately 40–80% [32]. Treatment with thiazolidinediones reduces hepatic steatosis by 30–50% by modulating insulin sensitivity and endocrine function of adipose tissue in T2DM [33]. However, metformin, an antihyperglycemic drug, improves hepatic insulin action without affecting liver lipid content [32]. In our study, there was a significant decrease in liver lipid content in linagliptin-treated DIO mice; liver fat content was reduced by up to 30% and insulin sensitivity was improved by up to 35%. Indeed, improved hepatic steatosis is the strongest predictor of improved glucose homeostasis. These data support the hypothesis that decreased lipotoxicity in response to linagliptin treatment may result in improved insulin sensitivity. Further studies comparing the effects of linagliptin with other glucose-lowering drugs on improved insulin sensitivity and reduced liver fat content may help to find the mechanisms underlying these beneficial effects of linagliptin treatment.

Shirikawa and coworkers (2011) recently investigated the effects of sitagliptin on adipose tissue inflammation and liver steatosis [23]. In β-cell-specific glucokinase haploinsufficient diabetic mice challenged with diets containing a combination of sucrose with either oleic or linoleic acid, they found that DPP-4 inhibition significantly prevented adipose tissue infiltration by macrophages, decreased the expression of plasminogen activator inhibitor-1 and prevented fatty liver [23]. In the liver, DPP-4 inhibition also decreased the expression of *SREBP-1c, SCD-1* and *FAS*, and increased the expression of *PPARα*. We therefore tested the hypothesis that linagliptin may have similar effects on adipose tissue biology and liver mRNA expression in our study. In accordance with Shirikawa et al [23], we found a significantly reduced number of macrophages infiltrating adipose tissue with 30 mg/kg/day linagliptin treatment, whereas the 3 mg/kg/d dose was not sufficient to reduce adipose tissue inflammation. Moreover, we found significantly reduced liver mRNA expression of *PTP1B* and *SOCS3* in the 30 mg/kg/day linagliptin treatment group. *PTP1B* has been implicated in the development of inflammation and insulin resistance associated with obesity during aging [34]. *SOCS3* is increased in inflammation and is thought to contribute to the pathogenesis of insulin resistance by inhibiting insulin signaling [35]. Collectively, the reduction of macrophage numbers in adipose tissue, as well as the decreased liver expression of *PTP1B* and *SOCS3* (for the latter also protein was reduced), may reflect the improved inflammatory status of these key insulin target organs with linagliptin treatment. These data suggest that decreasing inflammation at the organ level in mice treated with linagliptin 30 mg/kg/day may contribute to improved whole body insulin sensitivity. In addition, linagliptin (30 mg/kg/day dose only) significantly reduced *SREBP1c, SCD1* and *FAS* expression. These data are in accordance with the effects of sitagliptin treatment on liver gene expression [23]. However, with the study design used we cannot distinguish whether reduced *SREBP1c, SCD1* and *FAS* expression cause, or are merely markers of, a reduction in liver fat content. Further studies are required to find the potential direct effects of DPP-4 inhibition on these genes associated with hepatic steatosis. In our model system, we did not find significant effects of linagliptin on the expression of genes involved in fatty acid oxidation (*CPT1α* and *ACOX1*) and the *GLP-1 receptor* suggesting that alterations of these pathways are not primarily responsible for improved insulin sensitivity and reduced liver fat content in linagliptin-treated DIO mice.

In conclusion, although the current study design cannot distinguish whether significantly improved insulin sensitivity in response to long-term treatment with linagliptin is a direct or secondary effect, the data suggest that it may be the result of significantly reduced liver fat content in diet-induced hepatic steatosis and insulin resistance animal model. These results may further qualify the use of linagliptin in pathophysiological conditions of fatty liver diseases.
